# Effects of experience on recognition of speech produced with a face mask

**DOI:** 10.1186/s41235-022-00388-4

**Published:** 2022-05-26

**Authors:** Anne Marie Crinnion, Joseph C. Toscano, Cheyenne M. Toscano

**Affiliations:** 1 Department of Psychological Sciences, University of Connecticut, Storrs, USA; 2 Department of Psychological and Brain Sciences, Villanova University, Villanova, USA

**Keywords:** Speech perception, Perceptual learning, COVID-19, Face masks

## Abstract

Over the past two years, face masks have been a critical tool for preventing the spread of COVID-19. While previous studies have examined the effects of masks on speech recognition, much of this work was conducted early in the pandemic. Given that human listeners are able to adapt to a wide variety of novel contexts in speech perception, an open question concerns the extent to which listeners have adapted to masked speech during the pandemic. In order to evaluate this, we replicated Toscano and Toscano (PLOS ONE 16(2):e0246842, 2021), looking at the effects of several types of face masks on speech recognition in different levels of multi-talker babble noise. We also examined the effects of listeners’ self-reported frequency of encounters with masked speech and the effects of the implementation of public mask mandates on speech recognition. Overall, we found that listeners’ performance in the current experiment (with data collected in 2021) was similar to that of listeners in Toscano and Toscano (with data collected in 2020) and that performance did not differ based on mask experience. These findings suggest that listeners may have already adapted to masked speech by the time data were collected in 2020, are unable to adapt to masked speech, require additional context to be able to adapt, or that talkers also changed their productions over time. Implications for theories of perceptual learning in speech are discussed.

**Supplementary Information:**

The online version contains supplementary material available at 10.1186/s41235-022-00388-4.

## Introduction

In order to effectively perceive speech, listeners must adjust for contextual variability. This variability arises from a number of sources, including both talker-specific factors, such as their accent, and environmental factors, such as background noise. With experience, listeners show adaptation to novel contexts, a process known as perceptual learning (see Samuel and Kraljic 2009, for a review). As a result of the COVID-19 pandemic, listeners now have substantial experience with a new source of contextual variability: speech produced while the talker is wearing a face mask (hereafter, “masked speech”). While some listeners may have had prior experience with masked speech (e.g., healthcare workers; Radonovich et al. 2009), for most people, masked speech has presented a novel context during the pandemic. This raises questions about the efficacy of communication with masked speech (Mendel et al. 2008) and may contribute to hesitancy about wearing masks to limit the spread of the SARS-Cov-2 virus.

In previous work (Toscano and Toscano 2021, hereafter T&T), we evaluated the effectiveness of different types of face masks for speech communication. Those data were collected at an earlier point in the pandemic (July–August 2020) when listeners had less experience with masked speech. Here, we replicate that experiment with different listeners in a large sample meant to represent trends in the general population in order to investigate whether experience with masked speech led to improvements in recognition over time, shedding light on the extent and limits of perceptual learning in real-world contexts. Additionally, we consider how individual participants’ experiences with masks over the course of the year affected their ability to recognize masked speech. In the following sections, we briefly review previous work examining effects of face masks on speech recognition, as well as work examining perceptual learning in speech. Next, we present an experiment based on T&T and compare the current results to those found in that study.

### Effects of face masks on speech recognition

At first glance, face masks seem likely to impair speech recognition, both because they eliminate visual speech information and because they affect the acoustic signal. A number of previous studies have investigated the acoustic effects of face masks on speech and found that, as expected, masks affect the acoustic characteristics of speech sounds to some extent. In general, these studies have found that masks block spectral information above 1 kHz (Corey et al. 2020; Palmiero et al. 2016; Pörschmann et al. 2020), and some types of masks (e.g., cloth masks) have a larger effect than others (e.g., surgical masks; Bottalico et al. 2020; Corey et al. 2020; Toscano and Toscano 2021).

Because speech includes acoustic cues at frequencies affected by masks, some of these effects could influence listeners’ speech recognition accuracy. However, like the acoustic effects, impacts on speech recognition depend on the type of mask. Previous studies investigating the effects of surgical masks tend to find little to no effect on speech recognition (Mendel et al. 2008; Thomas et al. 2011). In contrast, studies investigating cloth masks have found a range of different effects. Some have shown more substantial effects on speech recognition (Bottalico et al. 2020; Brown et al. 2021), while others have found effects comparable to other types of masks (Magee et al. 2020) or have found little effect beyond the loss of visual information (Llamas et al. 2009). Studies examining N95 respirators have found a range of effects that depend on the specific mask (Radonovich et al. 2009).

T&T investigated how the type of mask and level of background noise affect listeners’ ability to recognize masked speech. Listeners heard sentences from the Hearing in Noise Test (HINT; Nilsson et al. 1994) produced while the talker was wearing either a surgical mask, N95 respirator, one of two types of homemade cloth masks, or no mask. Sentences were embedded in multi-talker babble at either a relatively high signal-to-noise ratio (SNR) of $+13$ dB or a relatively low SNR of $+3$ dB. We found that both the type of mask worn by the talker and the level of background noise affected listeners’ ability to accurately recognize the spoken sentences. At the high SNR, masks have only a small effect on speech recognition. In contrast, at the low SNR, some masks (the N95 respirator and the two cloth masks) led to poorer recognition compared with the no-mask condition.

Recent work has also identified other factors that influence listeners’ perception of masked speech. Brown et al. (2021) presented participants with videos of talkers with or without masks at various SNRs. They found that listening effort was greater with face masks, even when speech recognition accuracy was relatively similar across the mask and no-mask conditions at a high SNR. Interestingly, while background noise does affect perception of masked speech, other factors that can lead to listening difficulty, such as age do not seem to have such an effect. While older adults tend to perform worse overall on speech perception tasks, the relative impact of masks is generally consistent with what is observed in younger listeners (Brown et al. 2021). Hearing loss is another factor that can affect perception of masked speech, and listeners with hearing loss report greater difficulties perceiving masked speech in a variety of situations (Poon and Jenstad 2022; Saunders et al. 2021).

In addition, face masks can affect how the talker produces speech, which in turn, can impact speech recognition. Magee et al. (2020) found that talkers include more pauses in speech produced with a surgical or N95 mask, which could potentially serve as a compensatory mechanism to give listeners more time to comprehend speech. Similarly, clear speech produced with a mask does not result in decreased recognition. Cohn et al. (2021) found that when talkers produce clear speech while wearing a mask, listeners accurately recognize more words than when talkers produce clear speech without a mask. This result suggests that when talkers are aware of the need to speak more clearly (e.g., due to comprehension being more difficult because of the mask), they can compensate for the acoustic effects of the mask and are able to make their speech more intelligible. Not all effects of masks on speech production lead to compensation, however. McKenna et al. (2022) found that talkers’ vowel space is actually reduced with a mask, compared to without, and talkers report greater vocal effort. Hence, masked speech alters production in some ways that might make speech perception more challenging.

In sum, previous work demonstrates that, in relatively low levels of background noise (i.e., at high SNRs), the acoustic effects of masked speech produce only a small impact on speech recognition. Under more challenging listening conditions (low SNRs), face masks have a larger effect. Notably, differences in speech recognition observed for different types of masks are consistent with the acoustic effects that each specific mask type has on the speech signal. These results suggest that to the extent that listeners’ perception has improved during the pandemic with greater experience listening to masked speech, we may see the largest improvements in conditions with higher levels of background noise (where there is more room for improvement). However, talkers may be sensitive to the acoustic effects of masks and adjust their speech accordingly to overcome potential comprehension difficulties. Thus, over the past year, listeners may have primarily encountered masked speech in contexts where talkers were compensating for the acoustic effects of masks. In other words, *talkers* may have adapted to masked speech, even if listeners have not. If this is the case, we might see that listeners show a similar level of accuracy to listeners tested at an earlier point during the pandemic.

It is also possible that listeners’ adaptation to masked speech depends on the types of encounters that they have had over the past year. Work from Adjodah et al. (2021) suggests that implementation of mask mandates, for example, leads to greater mask adherence. As a result, listeners residing in an area with a mask mandate may have had more experience with masked speech. Similarly, individual listeners may have had more or less experience with masked speech over the past year depending on their daily activities. It is possible that more experience with masked speech leads to greater improvements in speech recognition, as it provides more opportunities for learning. We consider this possibility by looking at whether or not participants resided in a state with a mask mandate (using state policies listed by Adjodah et al. 2021) and self-reported measures of participants’ own frequency of mask use and the frequency of mask use by those they interacted with.

### Perceptual learning in speech

Before assessing whether perceptual learning occurred for masked speech during the pandemic, we first consider previous work on perceptual learning in speech more broadly. One of the most prominent approaches for studying these effects has been the lexically-guided perceptual learning paradigm. Learning in this paradigm occurs when a listener shifts a phonetic category boundary based on experience with an atypical pronunciation in a lexical context (Norris et al. 2003; Samuel and Kraljic 2009). For example, hearing an ambiguous sound between /$\int$/ and /s/ in contexts where lexical information disambiguates the sound as an /s/, such as *epi?ode* will eventually lead listeners to interpret the ambiguous sound as an /s/, since only *episode* is a word.

While perceptual learning using this approach has been demonstrated in a large number of experiments (Eisner and McQueen 2005, 2006; Jesse and McQueen 2011; Kraljic and Samuel 2005, 2006; Norris et al. 2003; Samuel and Kraljic 2009), it does not always occur for non-standard pronunciations. For example, Kraljic et al. (2008) showed that perceptual learning does not occur when atypical pronunciations can be attributed to an external source. When the talker is speaking with a pen in their mouth, for example, listeners attribute variation in the speech signal to this temporary state and do not update their representations of speech sounds for that talker. Follow-up work from Kraljic and Samuel (2011) suggests that being able to attribute variation in the speech signal to something external leads to a separate representation that the listener can access. Thus, if listeners attribute differences in masked speech to the presence of a face mask, they may acquire new representations that allow them to recognize masked speech effectively, while retaining representations for perceiving speech produced without a face mask.

However, Liu and Jaeger (2018) suggest that listeners do maintain some information about ambiguous tokens that could be attributed to an external cause such as a pen in the mouth (as opposed to ignoring it or completely treating it as a separate representation). Their work suggests that listeners may attribute some of the atypicality heard from sounds produced with a pen in the mouth to characteristics of the talker if it is followed by evidence of more atypicality present without the pen. However, note that all of these studies concern talker-specific perceptual learning. In contrast, with face masks, listeners likely have exposure to many different talkers producing masked speech, which may limit the generalizability of this work to the context of masked speech.

Background noise can also block lexically-mediated perceptual learning, a factor that may be relevant for masked speech given the differences observed at high versus low SNRs. Zhang and Samuel (2014) found that learning did not occur when speech was presented in background noise, even though listeners could accurately recognize the words (confirmed via a separate transcription task). In contrast, under conditions of cognitive load, perceptual learning was still observed. Thus, the lack of learning in background noise is not simply due to the difficulty of the task. Instead, Zhang and Samuel posit that it may not be optimal for the perceptual system to attribute differences in pronunciation to the talker when they can be attributed to signal degradation.

### Adaptation to novel acoustic distributions

The lexically-guided perceptual learning paradigm demonstrates listeners’ flexibility in adapting to novel sources of contextual variability, as well as some of the limitations of this flexibility. However, this approach involves highly specific manipulations of acoustic cues (e.g., making a sound ambiguous between /s/ and /$\int$/) in specific lexical contexts. While masked speech might affect acoustic cues to certain phonological distinctions more than others (Fecher and Watt 2011), the overall acoustic effect of masks is that they dampen higher-frequency sounds. This could lead listeners to adapt to masked speech by more heavily weighting low-frequency acoustic cues, an approach consistent with cue-integration models of speech perception that weight cues based on their statistical reliab﻿ility (Crinnion et al. 2020; Nearey 1990; Oden and Massaro 1978; Toscano and McMurray 2010).

Perceptual learning has been demonstrated for these types of changes as well, using paradigms in which the statistical distribution of acoustic cues is altered (Clayards et al. 2008; Munson 2011). For example, Clayards et al. (2008) found that listeners are sensitive to the amount of variation within a perceptual category; when voice onset time (VOT) values vary within phonetic categories either over a narrow or wide range, listeners adjust their categorization of sounds varying in VOT accordingly (i.e., they show a steeper categorization function when less variation is present and a shallower categorization function when more variation is present). Listeners are also able to adapt to shifts in the mean VOT value of speech sounds distinguishing phonetic categories, showing a shift in their category boundaries for specific talkers without any explicit instruction about differences between the talkers (Munson 2011).

Experience with other types of acoustic manipulations, such as spectral shifts, can also result in learning (see Samuel and Kraljic 2009, for an overview). For example, Peelle and Wingfield (2005) found that listeners improved with experience listening to noise vocoded speech that was spectrally-shifted downwards. Other studies have found similar adaptation to shifted spectral information (Rosen et al. 1999; Stacey and Summerfield 2007). Thus, listeners may be able to improve their recognition of masked speech by downweighting higher-frequency spectral cues that are missing from the speech signal. On the other hand, there are likely to be limits to such adaptation: to the extent that masks reduce the number of informative cues in the signal, adaptation may yield little to no improvement in speech recognition.

### Current study

The current study examines whether listeners have improved over the past year in perceiving speech produced with face masks, providing a real-world test case of perceptual learning. Previous work suggests several possible effects that experience with masked speech may have on listeners’ speech recognition. First, because face masks block some of the acoustic information in the speech signal, we may see no improvements. In other words, it may be that listeners at an earlier point in the pandemic were already recognizing masked speech as best as they could. Alternatively, because listeners can adapt to systematic changes in the acoustic distribution of speech, as seen with spectrally-shifted sounds (Peelle and Wingfield 2005), we may see improvements over time.

Second, previous studies demonstrate that masks have a greater effect on speech recognition in higher levels of background noise. Thus, we may see the greatest improvement in speech recognition under these conditions. On the other hand, it is possible that listeners have not had much experience with masked speech in higher levels of background noise, especially with restrictions on occupancy in crowded places throughout the pandemic, which in turn, may limit the amount of learning that would have occurred in these contexts. Likewise, the fact that previous work has found that perceptual learning may not occur for speech in noise (Zhang and Samuel 2014) suggests that, even if listeners have had experiences with masked speech in noise, they may not have improved over time.

To evaluate these possibilities, we replicate the experiment from T&T to see whether listeners tested in the summer of 2021 perform better than listeners tested in the summer of 2020 on the same task. In addition, to further explore whether experience with masked speech impacts recognition, we investigated whether self-reported degree of mask wearing during interactions with others affected their recognition of masked speech. We also explore whether participants living in a state that implemented a public mask mandate, which provides an indirect measure of the extent of encounters with masked speech, outperformed those who live in a state that did not implement a public mask mandate.

## Method

All design and analysis procedures were preregistered and are available at: https://aspredicted.org/4bh7d.pdf. Other than the addition of follow-up questions regarding the use of face masks and state of residence, the methods were identical to T&T.

### Participants

A total of 200 participants (82 female, mean age: 38 years) completed the study. Subjects were recruited from Amazon’s Mechanical Turk service (Seattle, WA) and were compensated for their time. This study was approved by the Villanova University Institutional Review Board.

### Stimuli

Stimuli consisted of the same audio recordings used in T&T, which were 20 sentences in two lists from the HINT (Nilsson et al. 1994). These sentences were recorded by two talkers (authors CMT [female, Talker 1] and JCT [male, Talker 2]) with five mask conditions (no mask, surgical mask, pleated cloth mask, fitted cloth mask, and N95 respirator). Six-talker (3 female, 3 male) babble noise was applied to these recordings with two different SNRs ($+3$ vs. $+13$ dB). As in T&T, listeners heard one HINT list at a specific SNR and the other list at the other SNR (with the order counterbalanced across subjects). Within each list, subjects heard one sentence for each combination of talker and mask, with the assignment of experimental conditions to specific sentences determined using a Latin Square design, resulting in a total of 20 sentences per subject. Stimuli were blocked by SNR, and trial order within each HINT list was randomized.

### Procedure

The procedure followed that of T&T. Participants completed the experiment using the Qualtrics (Provo, UT) online platform. First, participants provided informed consent, completed a brief demographic information form, and provided information about the type of headphones they were wearing. Participants were then instructed to listen to sentences and type what they heard. Two practice trials (one produced by each talker) with no background noise were presented first to ensure that listeners could accurately hear the stimuli, and then participants completed the two blocks of ten trials each with a break in between.

After completing the main task, participants completed five additional survey questions (these questions were not present in the original T&T study). The first four questions asked participants to reflect on their interactions with other people in the past month and in the past year, and to rate on a scale from zero (never) to ten (always) (1) how often the other person was wearing a mask in the past month, (2) how often the participant was wearing a mask in the past month, (3) how often the other person was wearing a mask in the past year, and (4) how often the participant was wearing a mask in the past year. The final question asked participants to select the state in which they currently reside.

### Data analysis

Subjects were excluded from analysis following the same criteria as T&T: (1) if they self-reported non-normal hearing (N = 1), (2) if they made more than 50% errors in the practice trials (N = 18), or (3) if they did not provide valid responses on at least 50% of trials (N = 2). These criteria were designed to exclude participants who reported non-normal hearing status, as well as those whose practice performance indicated poor hearing and/or a lack of attention to the task. We thus included 179 subjects in our final sample. Individual trials were excluded from analysis if the response time exceeded 60 s. A total of 3559 trials were included in the analyses. Trials were scored as the number of correct words in each sentence, with alternate responses listed in the HINT included as correct as well. Words were scored as correct regardless of position in the sentence, and alternate spellings were accepted for the following words: *they’re* (*their* and *there* accepted) and *two* (*2*, *too*, and *to* accepted).

All statistical analyses were implemented as mixed-effects logistic regressions using the lme4 (Bates et al. 2015) package in R (R Core Team 2020). For the main analysis of the current experiment, the model structure and coding scheme exactly followed that of T&T, with fixed effects of mask type, talker, SNR, and their interactions, along with by-subject and by-item (HINT sentence) random slopes. Mask type was effect coded, with the no-mask condition as the reference level. To further analyze differences between the current data set (Experiment 2, collected in 2021) and the data from T&T (Experiment 1, collected in 2020), a second model was run with an additional fixed effect of Experiment (effect coded: $\hbox {Experiment 1} = -0.5$, $\hbox {Experiment 2} = 0.5$) and its interactions with the other factors.

Next, we analyzed whether participants’ self-reported experience with masked speech influenced recognition accuracy and effects of face masks by using responses to the mask encounter questions. These responses were numerically-coded, z-scored, and included as a fixed effect in the model, along with fixed effects of mask type, SNR, talker, and their interactions. Lastly, to analyze whether listeners’ accuracy varied based on whether or not the state they reside in implemented a public mask mandate at some point prior to the date of data collection, we coded each participant’s state as either “mandate” or “no mandate”, with mandate meaning the state implemented a public mask mandate at some point due to COVID-19 (based on data from Adjodah et al. 2021). This variable was numerically coded ($\hbox {No mandate}=-0.5$, $\hbox {Mandate} = 0.5$) and centered, and included as a fixed effect in a model, along with effects of mask type, SNR, talker, and their interactions.

## Results

### Speech recognition performance in current experiment

**Fig. 1:**
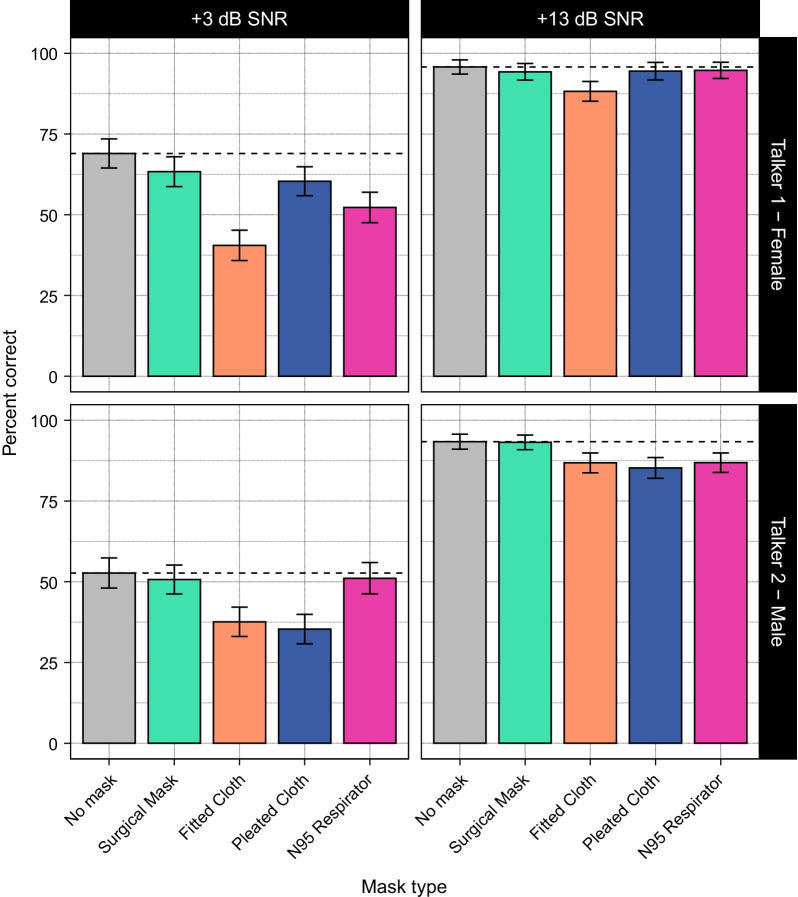
Average performance by talker, mask, and SNR from the current dataset (collected in 2021). Similar to Toscano and Toscano (2021), listeners were more accurate in the high SNR condition, and more accurate for Talker 1. Compared to the no-mask condition, the surgical mask yielded the best performance, followed by the N95 respirator, and the two cloth masks. Dashed lines indicate the average performance for the no-mask condition in each panel. Error bars indicate 95% confidence intervals

First, we examined how the current set of participants performed on the speech recognition task. Figure 1 shows listeners’ speech recognition accuracy as a function of mask type, SNR, and talker. Similar to T&T, we found that listeners were more accurate in the high SNR condition (mean accuracy: 91.2%) compared to the low SNR condition (mean accuracy: 51.3%). We also found that listeners were more accurate when listening to Talker 1 (75.4% correct overall, with 57.2% correct in the low SNR condition and 93.5% correct in the high SNR condition) compared to Talker 2 (67.3% overall, 45.5% in the low SNR condition, 89.0% in the high SNR condition).

Mask type also affected speech recognition. Accuracy in the no-mask condition (77.6% overall, with 60.7% in the low SNR condition and 94.4% in the high SNR condition) was similar to accuracy with the surgical mask (75.4% overall, with 57.1% in the low SNR condition and 93.6% in the high SNR condition) and the N95 respirator (71.3% overall, with 51.7% in the low SNR condition and 90.9% in the high SNR condition). In contrast, accuracies for the fitted cloth mask (63.3% overall, with 39.2% in the low SNR condition and 87.4% in the high SNR condition) and the pleated cloth mask (69.0% overall, with 47.9% in the low SNR condition and 89.8% in the high SNR condition) were somewhat lower than the no-mask condition. Overall, the pattern of results was similar to that found in T&T, except that accuracy in the low SNR condition was somewhat higher across all conditions (including the no mask condition).

To validate these results statistically, we fit a logistic mixed-effects model to the data (see Method for details about model structure). The model revealed a main effect of SNR (*b* = 4.25, *SE* = 0.13, *z* = 32.16, *p* < 0.001), demonstrating that listeners were more accurate at the higher SNR. There was also a main effect of talker (*b* = 1.04, *SE* = 0.20, *z* = 5.34, *p* < 0.001), demonstrating that listeners were more accurate with speech produced by Talker 1. In contrast to T&T, we did not observe an SNR $\times$ talker interaction.

The model also revealed main effects of both of the cloth masks (fitted mask: *b* = −1.33, *SE* = 0.20, *z* = −6.66, *p* < 0.001; pleated mask: *b* = −0.75, *SE* = 0.21, *z* = −3.64, *p* < 0.001), meaning that for speech produced with either of the cloth masks, listeners recognized fewer words correctly than for speech produced without a mask. There was also a pleated mask $\times$ talker interaction (*b* = 0.92, *SE* = 0.46, *z* = 2.01, *p* = 0.04). Follow-up analyses revealed a significant effect for the pleated mask for Talker 2 (*b* = −0.80, *SE* = 0.19, *z* = −4.29, *p* < 0.001) and a marginal effect for Talker 1 (*b* = −0.47, *SE* = 0.26, *z* = −1.77, *p* = 0.08). In contrast to T&T, we did not find an interaction between mask and SNR for either cloth mask.

There was also no main effect for the N95 respirator, which differed from T&T and suggests that listeners’ performance for this mask may have improved. However, like T&T, we found a three-way interaction between N95 respirator, SNR, and talker (*b* = 2.01, *SE* = 0.46, *z* = 4.35, *p* < 0.001). Follow-up analyses revealed that, at the high SNR, accuracy was significantly lower for the N95 respirator for Talker 2 (*b* = −1.11, *SE* = 0.25, *z* = −4.40, *p* < 0.001), but effects were nonsignificant for Talker 1. Conversely, at the low SNR, effects were nonsignificant for Talker 2, but accuracy was significantly lower for Talker 1 (*b* = −1.09, *SE* = 0.34, *z* = −3.18, *p* = 0.001). This differs from the pattern for the three-way interaction observed by T&T for the N95 respirator, and the lack of a two-way interaction between the N95 respirator and SNR also differs from T&T. Overall, the effects for the N95 respirator appear to be idiosyncratic to each experiment, making it difficult to draw conclusions about any improvements in accuracy for this mask.

There were no main effects or interactions for the surgical mask, meaning that performance was not statistically different from the no-mask condition. This is the same result as T&T, providing additional evidence that the acoustic effects of the surgical mask have little to no effect on speech recognition.

### Changes in masked speech recognition during the pandemic

**Fig. 2:**
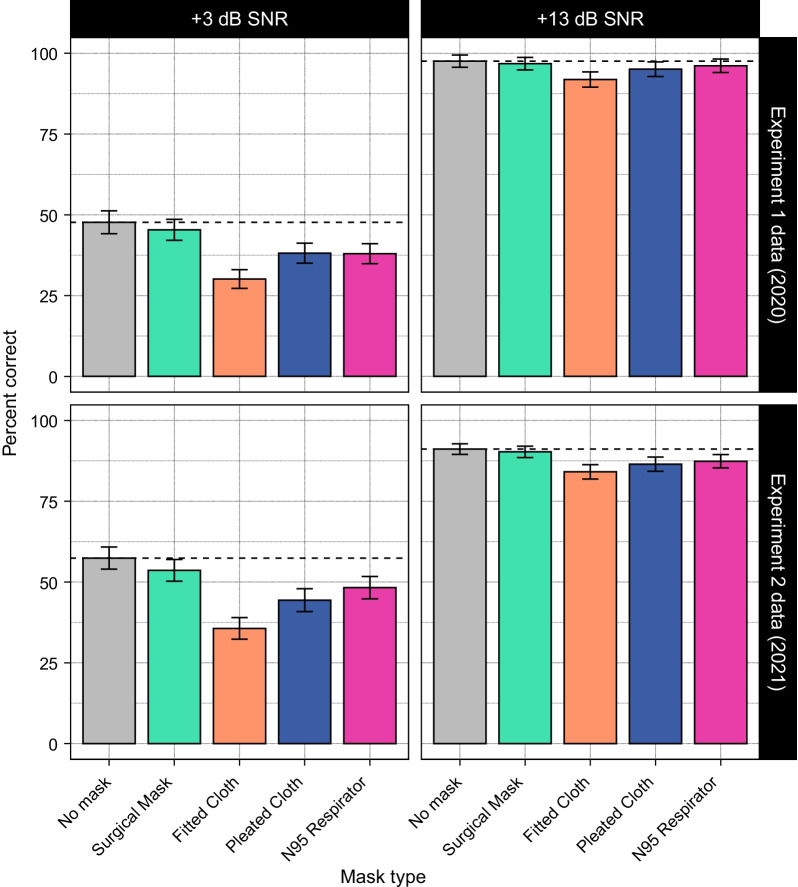
Average performance by mask type and SNR, for each year of data collection. Overall, trends are similar between the two datasets, with participants from the 2021 experiment performing better in the low SNR condition than participants from 2020. Dashed lines indicate the average performance for the no-mask condition in each panel. Error bars indicate 95% confidence intervals

In order to directly compare listeners’ performance in 2021 to performance in 2020, we combined these data with the data from T&T. Figure 2 shows speech recognition accuracy as a function of mask type and SNR for each dataset (collapsed across talker). Overall, the effects of each mask on listeners’ accuracy were similar in both experiments, though listeners in the current experiment were more accurate in the low SNR condition relative to listeners in T&T and performance was slightly worse in the high SNR condition.

These effects were validated statistically using a logistic mixed effects model, with an additional fixed effect of experiment (Experiment 1, T&T data collected in 2020; and Experiment 2, current dataset collected in 2021) and all its interactions with the other fixed effects (SNR, talker, and mask type; see Method for details on the structure of the model). Of particular interest are any effects involving experiment that interact with effects of mask type. The presence of such effects would indicate that performance differed in some way across the two time points as a function of mask.

We found a main effect of experiment ($b = 0.51$, ${\textit{SE}}=0.18$, $z=2.90$, $p=0.004$), such that participants run in 2021 were more accurate across all conditions than participants run in 2020 (Experiment 1 overall accuracy: 64.5%; Experiment 2 overall accuracy: 71.3%). We also found a significant interaction between experiment and SNR ($b=-1.15$, ${\textit{SE}}=0.21$, $z=-5.47$, $p<0.001$). Follow-up analyses revealed that at the low SNR, accuracy differed between the two experiments ($b=0.84$, $\textit{SE}=0.14$, $z=5.93$, $p<0.001$), with listeners in Experiment 2 showing higher accuracy (51.3%) compared to listeners in Experiment 1 (36.5%). However, there was no significant difference between the two experiments at the high SNR (Experiment 1 accuracy: 92.1%; Experiment 2 accuracy: 91.2%).

No other interactions involving experiment were significant. This suggests that the effects of the individual masks on listeners’ performance did not change over the course of the pandemic.

### Effects of self-reported experience listening to masked speech

Next, we examined whether speech recognition performance differed as a function of how often listeners interacted with talkers who were wearing a face mask in the past month and in the past year (all data were collected in June 2021). This may provide a more fine-grained estimate of the extent to which experience with masked speech influenced speech recognition. We asked participants to rate on a scale from 0 to 10 how often their interactions involved the other person wearing a mask (0 indicates other talkers never wore a mask, and 10 indicates they always wore a mask). For interactions in the past year, listeners reported a mean estimate of 7.7 (SD: 1.9), and for interactions in the past month, listeners reported a mean estimate of 5.7 (SD: 2.6). We also asked participants to rate on a scale from 0 to 10 how often they wore a mask in their interactions with others. For interactions in the past year, listeners reported a mean estimate of 8.2 (SD: 2.4), and for interactions in the past month, listeners reported a mean estimate of 6.4 (SD: 3.5). For purposes of examining whether experience over the past year with masked speech affected speech recognition, we focus on the reports of mask wearing over the past year.

**Fig. 3:**
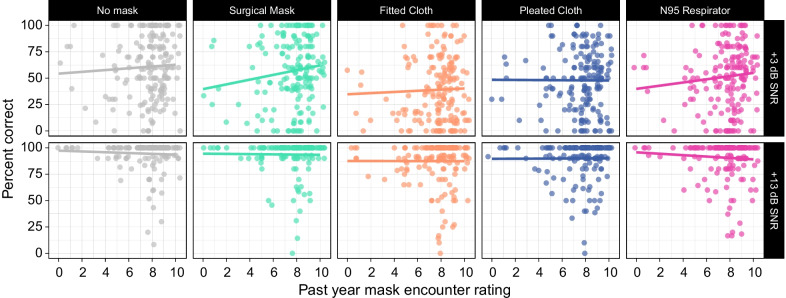
Average performance by mask type and SNR as a function of self-reported frequency of encounters with masked individuals in the past year. Trend lines represent simple linear regressions. Although there is a positive trend at the low SNR for the surgical mask and N95 respirator, these effects were not statistically significant

**Fig. 4:**
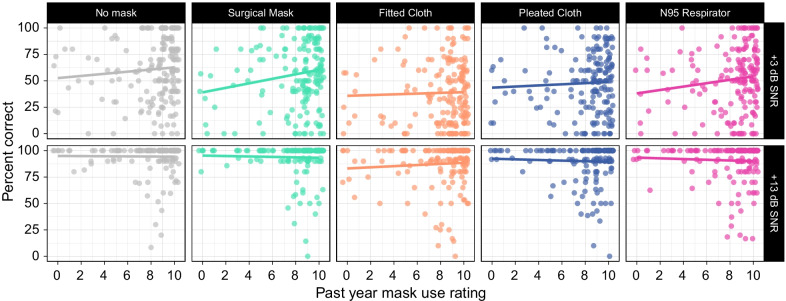
Same as Fig. 3 for self-reported frequency of the participant’s own mask use in the past year

Figure 3 shows the correlation between ratings of others’ mask use and speech recognition accuracy as a function of mask type and SNR, and Fig. 4 shows the same for self mask use. We see similar trends for both measures of mask use. At the high SNR, mask encounter rating did not correlate with performance on the speech recognition task for any of the face mask conditions. At the low SNR, a slight positive trend is observed for the surgical mask and N95 respirator.

These effects were assessed using a mixed effects model with mask encounter responses as an additional fixed effect, along with the other fixed effects in the original model (SNR, mask type, talker) and all interactions. We found no significant effects or interactions involving mask encounter for the model that included encounters with masked individuals or for the model that included personal mask wearing. This suggests that performance did not differ based on mask experience in the past year. Models run using the questions about mask experience in the past month showed similar findings; namely, there were no interactions between mask experience and mask type. The model that included the question about experience with others’ mask use in the past month revealed a main effect of mask experience ($b=-0.29$, $\textit{SE}=0.12$, $z=-2.41$, $p=0.016$), with listeners who reported greater mask experience performing worse.

### Performance as a function of state-level public mask mandates

Finally, we examined whether speech recognition performance differed across individuals residing in different states. Specifically, we examined differences in performance as a function of whether or not the listener’s state of residence implemented a public mask mandate during the pandemic (based on data from Adjodah et al. 2021).

**Fig. 5:**
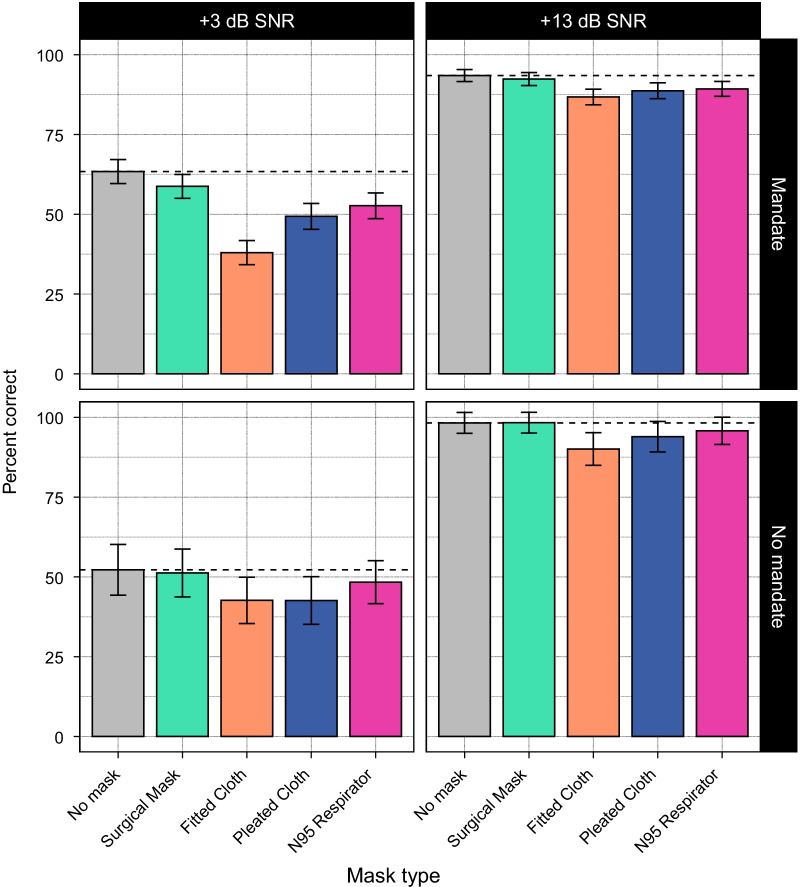
Average performance by mask and SNR, grouped by whether participants’ state of residence implemented a public mask mandate during the pandemic ($\hbox {no mandate}=41$ participants; $\hbox {mandate}=138$ participants). There were no differences as a function of mask mandates. Dashed lines indicate the average performance for the no-mask condition in each panel. Error bars indicate 95% confidence intervals

Forty one participants in the final dataset resided in a state without a public mask mandate, and 138 resided in a state with a mandate. Figure 5 shows speech recognition performance for each mask type and SNR (collapsed across talker) as a function of whether listeners resided in a state with a public mask mandate. Overall, performance is similar for both groups of listeners. A mixed effects model was run with fixed effects for talker, SNR, mask type, and public mask mandate (numerically coded, with $\hbox {No mandate} = -0.5$ and $\hbox {Mandate} = 0.5$, and scaled). No effects involving public mask mandate were significant, indicating that speech recognition accuracy did not vary based on whether or not the listener lived in a state with a public mask mandate. Given that public mask mandates have been shown to increase mask adherence (Adjodah et al. 2021), this suggests that listeners who were more likely to encounter masked speech during the pandemic did not show better performance than those who were not.

## Discussion

Overall, the effects of masked speech in the current experiment were similar to those observed by T&T. We replicated differences in speech recognition based on talker (Talker 1 was more intelligible than Talker 2) and SNR (performance was substantially better at the high SNR). Speech recognition also varied as function of mask type, with the two homemade cloth masks affecting performance the most, and no statistically significant effect of the surgical mask. When directly comparing these data (collected in June 2021) to the data reported in T&T (collected in July–August 2020), we find no differences in terms of performance with masked speech. While participants in the current sample were overall more accurate, particularly in the low SNR condition, these findings suggest that people did not learn to adapt to masked speech over the course of the pandemic. We note that the two datasets were collected with different participants. Hence, this study was not designed to test learning by specific individuals. However, even when considering subjects’ individual experiences with masks, we still did not find differences in performance with masked speech, again supporting the idea that adaptation to masked speech did not change with increased mask experience over the course of the pandemic.

Given the many other contexts in which listeners display perceptual learning in speech, why did we not see improvement in recognition of masked speech after nearly a year of experience with it? We discuss four possibilities: (1) listeners had already adapted to masked speech in T&T, (2) listeners cannot adapt to masked speech, (3) listeners need explicit information about context (e.g., a visual indicator that the talker is wearing a mask) to adapt to masked speech, or (4) talkers’ adjusted for effects of masks over the course of the pandemic (particularly in noisy contexts), so listeners did not have the opportunity to adapt to the type of speech produced by the talkers in our experiment.

### Possibility 1: listeners had already adapted

One possible explanation for our findings is that, rather than listeners failing to adapt, they actually adapted quite quickly to masked speech and reached a peak performance level prior to data collected in the summer of 2020. Indeed, many previous perceptual learning studies have demonstrated adaptation over the course of a single experimental session, and even a few minutes of exposure is sufficient for listeners to show adaptation to non-native speech (Clarke and Garrett 2004). It is interesting to note, however, that neither self-reported frequency of encounters with masked individuals, nor the public mask mandate policies of the state in which participants resided affected performance with masked speech. These results further suggest that perceptual learning either did not occur in the context of masked speech or learning occurred prior to July–August 2020.

### Possibility 2: listeners cannot adapt to masked speech

Another possibility is that listeners simply *cannot* learn or adapt to masked speech. As demonstrated by the acoustic analyses in T&T and other studies (Bottalico et al. 2020; Corey et al. 2020; Palmiero et al. 2016; Pörschmann et al. 2020), masks act as a low-pass filter of the speech signal. Thus, higher-frequency spectral cues may be either inaudible in masked speech or more susceptible to noise. While listeners can adapt to spectral changes in the speech signal (Peelle and Wingfield 2005; Rosen et al. 1999; Stacey and Summerfield 2007), there may simply not be enough information in lower-frequency cues to compensate for the loss of information caused by the mask. Similarly, listeners could treat the acoustic effects of the mask as noise, which may block perceptual learning (Zhang and Samuel 2014).

### Possibility 3: listeners require additional context to adapt to masked speech

Third, listeners may need additional information to show improvements in recognition of masked speech. For instance, Kraljic and Samuel (2011) suggested that when speech is produced with a pen in the talker’s mouth, listeners learn a separate representation for these sounds. If this is the case, then masked speech may exist as a separate representation upon which listeners can learn. Critically, Kraljic et al. (2008) found that listeners did not appear to learn after just hearing ambiguous pronunciations produced by a speaker with a pen in their mouth. However, listeners were tested on audio-only speech. It is possible then, that listeners were not able to access the correct representation (i.e., the representation for pen-in-mouth speech) due to the lack of audiovisual information in testing. Likewise, it is possible that speech produced with a pen in the mouth exists as a separate representation, but not one on which listeners adapt. Encountering a speaker with a pen in their mouth is an infrequent occurrence, and one that likely would not feasibly last for a long period of time. Masked speech, however, has been a more frequent and longer-duration external modifier of speech. Thus, this type of representation (i.e., speech produced with a mask) may be something that could be learned.

In the current experiment, stimuli were only auditory and listeners were not given any additional cues that some speech was produced with a mask. While this allows us to evaluate the acoustic effects of masked speech independently of any effects caused by loss of visual speech information, it also means that listeners did not have an explicit visual cue that the talker was wearing a face mask. Thus, the lack of learning we observed may be due to a lack of information about the context in which the speech was produced. Perhaps listeners were unable to access the learned (separate) representation of masked speech simply because there was not enough information. From a practical standpoint, this would be more promising in terms of listeners’ performance with masked speech, since many of their encounters with masked speech over the past two years most likely had a visual component. Thus, in real-world settings listeners may perform better than in the current experiment. This also suggests a limitation of perceptual learning, indicating that an explicit cue is sometimes needed for listeners to adapt to novel contexts.

Studies using audio-visual stimuli, such as Brown et al. (2021), may provide important insights into these issues. Future studies should also explore lab-based perceptual learning with masked speech to see whether or not unambiguous cues about masked speech (e.g., a visual indicator) could help elucidate which types of information aid in accessing specific learned representations or result in learning.

### Possibility 4: talkers adapted to speaking with a mask

Finally, it is possible that talkers adapted their production of masked speech during the course of the pandemic. This would make it less necessary for listeners to adapt to masked speech in order to recognize it accurately. It would also mean that the stimuli used in the current study, which were the same recordings as T&T and were produced at an early point in the pandemic, may not have been representative of the type of masked speech that listeners encountered later in the pandemic. As a result, listeners may have shown similar performance because they had little experience with this type of “unadapted” masked speech.

Evidence that talkers change their speech while wearing a mask further supports this possibility. Cohn et al. (2021) demonstrated that, when talkers produce clear speech while wearing a mask, they are more intelligible than they are for either clear or conversational speech produced without a mask. If talkers implicitly adopted the use of clear speech during the pandemic, listeners may have found our stimuli more challenging and less intelligible than the masked speech they were used to hearing. Future work should examine how masked speech production changes over time. On the one hand, talkers may be sensitive to the acoustic effects of masks and hence might try to speak clearly and may compensate even more over time to help listeners. On the other hand, as listeners perhaps adapt to masked speech, talkers may relax their efforts to speak clearly, which may cancel out improvements due to listener adaptation.

We also note that talkers change their speech in the presence of background noise, a phenomenon known as the Lombard effect (Lombard 1911). The sentences in the current experiment were presented in multi-talker babble noise, but they were recorded in quiet. Thus, the addition of noise to these sentences may not have reflected natural productions in noisy environments, where talkers might try to compensate for both the effect of background noise and the effects of the mask, perhaps by speaking louder or slower. We note, however, that Bond et al. (1989) did not find significant acoustic changes in talkers’ speech produced while wearing oxygen masks in noisy backgrounds compared to quiet backgrounds. Nonetheless, future research should explore exactly how background noise may affect masked speech production.

## Conclusions

In summary, we found that performance with masked speech did not change after almost a year of mask use. Notably, experience with mask wearing (both by the participant and those that the participant interacted with) did not influence performance. These results suggest that either listeners cannot adapt to masked speech, that they had already adapted to masked speech very early on in the pandemic, that they require additional context (e.g., audiovisual information) in order to access learned representations, or that talkers’ speech production also changed during the course of the pandemic. Future work should explore the exact nature of representations that can be learned in order to further distinguish between these possibilities. The extent to which learning can occur with masked speech remains an open question that informs theories of perceptual learning more broadly. By exploring questions of adaptation to various types of contexts, we can better understand what constitutes the representations upon which listeners build models of perceptual space.

## Supplementary Information

**Additional file 1:** Data file from the experiment.

**Additional file 2:** Script for statistical analyses.

## Data Availability

All data analyzed during this study along with analysis code are included in the Additional files 1 and 2 of this article.
